# An Unusual College Experience: 16-Month Trajectories of Depressive Symptoms and Anxiety among Chinese New Undergraduate Students of 2019 during the COVID-19 Pandemic

**DOI:** 10.3390/ijerph20065024

**Published:** 2023-03-12

**Authors:** Lili Liu, Jianbin Chen, Shunwei Liang, Xiaodan Peng, Wenwen Yang, Andi Huang, Xiayong Wang, Fang Fan, Jingbo Zhao

**Affiliations:** 1 Department of Psychology, School of Public Health, Southern Medical University, Guangzhou 510515, China; jackliull@outlook.com (L.L.); champagne_joe@126.com (J.C.);; 2 Mental Health Education and Counseling Center, Guangzhou Academy of Fine Arts, Guangzhou 510260, China; 3 Psychological Counseling Center, Department of Student Affairs, Yunnan University of Chinese Medicine, Kunming 650500, China; 4 Key Laboratory of Brain, Cognition and Education Sciences, Ministry of Education, Guangzhou 510631, China; 5 School of Psychology, Center for Studies of Psychological Application, Guangdong Key Laboratory of Mental Health and Cognitive Science, South China Normal University, Guangzhou 510631, China; 6 Mental Health Center, School of Public Health, Southern Medical University, Guangzhou 510515, China

**Keywords:** depression, anxiety, trajectories, new undergraduate students, latent growth mixture, Chinese students

## Abstract

Background: This study examines the trajectories of the mental health conditions of 13,494 new undergraduate students who enrolled in 2019 in China from the beginning of the pandemic to the local recurrence of the pandemic, and found factors which may be associated with diverse trajectories. Methods: The trajectories of depression–anxiety outcomes were modeled using the growth mixture model. The multinomial logistic regression model was used to identify variables associated with different trajectory groups. Results: Both depression and anxiety in the new college students slightly increased during the 16-month period. The slopes of depression and anxiety were lower after the local outbreak. From the trajectories of depression and anxiety, five heterogeneous groups were identified: low–stable (64.3%), moderate–increased (18.2%), high–stable (11.1%), recovery (4.5%), and rapid–increased (1.8%). Environmental, somatic, and social factors were used to differentiate the low–stable group from the other groups. We found that college students with female gender, more conflict with parents, and feelings of loneliness during the pandemic were more likely to enter a high stability trajectory compared to a recovery trajectory. Conclusion: Most participants showed a stable mental health status, while others experienced deteriorating or chronic mental health problems, especially those who had sleep disturbances, less social support before the pandemic, or conflicts with parents during the pandemic. These students may need additional support and monitoring from college mental health providers to improve their wellbeing.

## 1. Introduction

Since the end of 2019, the COVID-19 pandemic has spread all over the world. People’s daily lives have been deeply changed by it [1]. Due to the variants of SARS-CoV-2, the infectivity and virulence of the virus continued to change. The Delta variant of SARS-CoV-2 led to a local outbreak in Guangdong Province, China, on 21 May 2021, resulting in the implementation of strict prevention measures. A new variant of concern (VOC), Omicron, was reported on 24 November 2021, forcing many countries to restart strict anti-pandemic measures to prevent it from being wildly spread.

Meanwhile, mental health problems during the home confinement period of COVID-19 have become a global issue [2]. The data from China show that the mental health of the general population was significantly affected during the peak of the COVID-19 pandemic [3]. A systematic review of the literature found increased depression symptoms during the COVID-19 outbreak [4]; however, over the same period, another review found only a small-to-negligible deterioration of mental health symptoms [5]. Social isolation may increase loneliness during periods of high prevalence of COVID-19. The effects of this loneliness include depression, sleep problems, and social vigilance [6]. We are also concerned about how long the impact of the COVID-19-related social isolation on mental health conditions will last, and which factors will have a long-term impact and which will not.

College students—especially new students who enrolled in 2019—not only faced these enormous disruptions to daily life, but also encountered additional challenges, such as a shift to online learning and online exams for a whole semester. After in-person classes resumed, they had to stay at the campus most of the time. At the beginning of the outbreak, about 45% of students in China experienced probable acute stress, anxiety, or depressive symptoms [7]. A two-wave longitudinal survey showed a significant increase in the prevalence of mental health problems in students in China after the initial stage of the outbreak [8], and a nine-month longitudinal study of UK college students showed the same pattern [9]. However, the results of some studies did not show worsening mental health in university students during the COVID-19 pandemic [10,11], and a study of the immediate mental health status of Chinese college students when they returned to school showed no significant increase in the prevalence of psychiatric symptoms [12]. These opposing findings suggest that college students can have different mental health trajectories during the same period. Therefore, if we wish to know the trends in the mental health trajectories of university students, the use of longitudinal growth class analysis studies is most likely the best fit to answer the question. To our knowledge, longitudinal growth class analysis studies of mental health have mostly focused on nationally representative samples with limited amounts of time between the first and last wave (less than half a year), such as in the UK (four months) [13,14], Australia (three months) [15], and Germany (two months) [16]. Few researchers have focused their attention on the heterogeneous response to the pandemic of college students.

In addition, understanding the role of influencing factors in the trend is also of interest. Previous studies have found that the incidence rate of common mental health problems differs significantly due to sociodemographic characteristics such as gender, age, and living place [7]; pre-COVID-19 health status; previous mental and physical health problems [7,17]; attitudes towards COVID-19 [7,18]; lifestyle [19]; environmental factors, such as life events [20]; negative emotions during the lockdown, such as loneliness and despair [6,21]; somatic factors, such as sleep disturbance [22]; and social factors, such as social support and family function [23]. As the evidence begins to accumulate, it is safe to predict that mental health problems caused by the COVID-19 pandemic are not homogenous; as the authors of [13] have pointed out, some of these variables may continue to affect the mental health of students and some may not. It is important to find out which variables have a long-lasting effect and take action in future mental health services.

This study analyzes longitudinal data from five waves of representative samples of new students from 22 colleges from Guangdong Province, China, collected over 16 months from February 2020 to June 2021. We chose to examine the most common mental health problems: depressive symptoms and anxiety [15]. We tested four research questions related to the course of mental health difficulties during the start and subsequent easing of lockdown restrictions and the follow-up prevention measures which created uncertainty within China. The first research objective was to analyze the longitudinal impact of the COVID-19 pandemic on the mental health of new college students enrolled in autumn 2019. The second was to determine whether the local recurrence of the pandemic and tightened local anti-pandemic measures affect the mental health of local college students. The third was to identify if there were different longitudinal profiles of psychological distress over time during the COVID-19 pandemic. Additionally, fourth, we aimed to identify which factors, included characteristics, lifestyle, pre-COVID-19 health, COVID-19-related factors, environmental factors, negative emotions, somatic problems, and social factors, were associated with different longitudinal profiles.

## 2. Materials and Methods

### 2.1. Study Design and Participants

This longitudinal observational study was conducted on a large sample of college students from 22 colleges and universities in Guangdong Province, China. The study was conducted in five survey periods: 3–10 February 2020 (T1); 24 March to 3 April 2020 (T2); 1–15 June 2020 (T3); 10 September to 17 October 2020 (T4); and 10–18 June 2021 (T5).

Two criteria were used to screen the eligible subjects: a response time of less than 289 s (the total number of characters in the questionnaire was about 5280 and the maximum reading speed of Chinese characters was 1097/min [24]) and those who answered the entire questionnaire with the same answer.

A total of 13,494 newly enrolled 2019 undergraduates were included, who were selected from five longitudinal surveys and participated at least four times. T1: During the first outbreak phase of the pandemic, when students were confined to their homes, a total of 164,101 students (valid questionnaire: 88.3%) completed the first-wave survey. T2: A total of 148,343 students (valid questionnaire: 95.4%) completed the second-wave survey during the remission phase of COVID-19 (for an epidemiological evaluation of the first two surveys, see Ref. [8]) when students were still confined to their homes and the online study began. T3: 159,187 students (valid questionnaire: 95.7%) completed the third-wave survey during the normalization prevention phase when students were still confined to their homes and the online study continued. T4: 120,190 students (valid questionnaire: 97.5%) completed the fourth-wave survey after returning to school. T5: 93,413 students (valid questionnaire: 92.1%) completed the fifth-wave survey after Guangdong Province had new locally transmitted confirmed COVID-19 cases from mid-May 2021.

### 2.2. Procedures

A common normative communication was prepared for all 22 universities, which included the purpose, meaning, deadline, and mode of participation in the online survey. All students in the target universities were considered as potential participants and were asked to voluntarily participate in the survey via the networking platform (http://www.togx.cn/step_50.html, accessed on 18 June 2021). As the data collection was completed, we closed this website to prevent anyone from continuing to complete the survey. Only the students from the second year onwards were included in T4 and T5, as the senior students from T1 to T3 had graduated. (For more details, see Ref. [8].)

### 2.3. Measurements

#### 2.3.1. Depressive Symptoms

The Chinese version of the Patient Health Questionnaire (PHQ-9) was used to assess symptoms of depression [25]. It uses a 4-point scale ranging from 0 to 3, and consists of 9 items. A total score of 7 indicates probable clinical depression in college students [26]. The Cronbach alpha was 0.87 for T1, 0.90 for T2, 0.91 for T3, 0.92 for T4, and 0.92 for T5.

#### 2.3.2. Anxiety Symptoms

The Chinese version of the Generalized Anxiety Disorder Scale (GAD-7) was used to measure general anxiety symptoms [27], which consists of 7 items scored on a 4-point scale ranging from 0 to 3. As validated in a Chinese population, a cut-off total score of 7 indicates clinical levels of anxiety [28]. The Cronbach alpha was 0.91 for T1, 0.92 for T2, 0.94 for T3, 0.94 for T4, and 0.94 for T5.

#### 2.3.3. Covariates

Variables such as basic information about the participants, their lifestyle, psychological variables related to COVID-19 and negative emotions, social support, and family functioning were used as covariates. Specifically, information on each participant’s gender, age, living place, mental- and physical-health-related variables including major body disease, the receipt of psychological counselling, and the diagnosis of a mental illness. Lifestyle variables included smoking, alcohol consumption, and daily physical activity time. COVID-19-related psychological variables and negative emotions including life events and sleep disturbance, were measured by the Youth Self-Rating Insomnia Scale (YSIS) [29]. Social support was measured by the Multidimensional Scale of Perceived Social Support [30], and the family functioning was measured by Family APGAR [31]. Full details of covariates are provided in the online Supplementary Materials Section S1. 

### 2.4. Statistical Analysis

All statistical analyses were performed using SPSS 26.0 and Mplus 8.3 [32]. First, descriptive statistics were used to present the sample characteristics and detection rates of depression and anxiety. Paired t-tests were conducted to compare the slopes of one-class trajectories of mean depression symptoms and anxiety scores across T1 to T4 and T1 to T5. 

Second, multiclass solutions were compared statistically using the Akaike information criterion (AIC), the Bayesian information criterion (BIC), entropy, the Lo–Mendell–Rubin Likelihood ratio test (LMRT), the bootstrap likelihood ratio test (BLRT) [33], and the overall interpretability of solutions to determine the most parsimonious and clinically discriminative model. [34]. Entropy is a measure of the probability of belonging to a class and ranges from 0 to 1 (values closer to 1 are preferred); values above 0.80 are acceptable. The LMRT is a likelihood ratio test that provides a measure of the current mixture model (k) and a sample drawn with (k − 1) one latent class less than the current model [35]. The BLRT is a parametric bootstrap likelihood ratio test that compares the estimated model to a model with one less class than the estimated model [36]. Models were fitted to between 1 and 6 solutions of the latent classes. We used the model to estimate the means of the intercept and the slope functions for each class solution.

Finally, the multinomial logistic regression model was used to identify factors associated with different trajectory groups of depressive symptoms and anxiety in students. We used a three-step approach described by Kim et al. [37], which accounts for errors in classification and incorporates the classification uncertainties into the mixture model. It has also been shown to produce more accurate parameter estimates [38]. (See online Supplementary Materials Section S2 for the syntax of statistical analysis.)

## 3. Results

The mean age of the participants was 18.9 years (SD = 1.0). In total, 71% of the participants were female (*n* = 9583). Participants came from 31 of the 34 provinces in China.

Table 1 shows the detection rate of depression and anxiety at T1–T5 by gender. The overall detection rate of depression increased from 21.2% at T1 to 31.2% at T5 and the overall detection rate of anxiety increased from 10.4% at T1 to 19.7% at T5.

**Figure 1:**
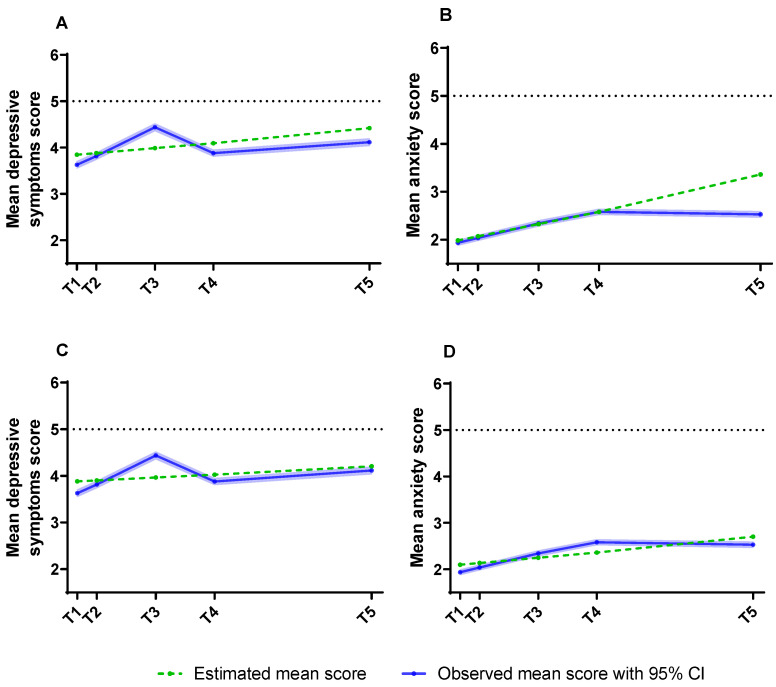
Simultaneous 1-class trajectories of mean estimated and observed depression symptoms and anxiety scores across different study timelines. (**A**,**B**): 7 months (T1–T4); (**C**,**D**): 17 months (T1–T5).

GMM was performed, estimating the fit for 1 to 6 classes that indicate the trajectory of both depressive symptoms and anxiety. Figure 1 shows the simultaneous one-class trajectories of mean depression symptoms and anxiety scores across the different study timelines. The mean slope of depressive symptoms was 0.036 (SE = 0.005, *p* < 0.001) for 7 months (T1–T4) and 0.020 (SE = 0.002, *p* < 0.001) for 16 months (T1–T5). Paired t-tests of the two slopes showed t = 6.880, *p* < 0.001, and Cohen’s d = 0.059, and the 95% CI for Cohen’s d ranged from 0.042 to 0.076. The mean slope of anxiety was 0.086 (SE = 0.004, *p* < 0.001) for 7 months (T1–T4) and 0.038 (SE = 0.002, *p* < 0.001) for 16 months (T1–T5). Paired *t*-tests of the two slopes showed *t* = 23.592, *p* < 0.001, and Cohen’s d = 0.203, and the 95% CI for Cohen’s d ranged from 0.186 to 0.220.

Table 2 shows the model fit indices for each of the six latent class solutions. Based on the fit indices, it was concluded that the five-class solution was the best-performing model (AIC = 1,011,782.25; BIC = 1,012,139.28). The entropy value was 0.861. The BLRT and LMRT were significant in the five-class models, but the LMRT was not significant in the six-class model, indicating that the model with the five classes is accepted in favor of the estimated model. The estimated classes were of acceptable size (11%, 18%, 5%, 2%, and 64% of the sample). The average latent class probabilities for inclusion in the each class for the five-class solution were 85.8%, 86.4%, 84.7%, 87.6%, and 95.9%.

**Figure 2:**
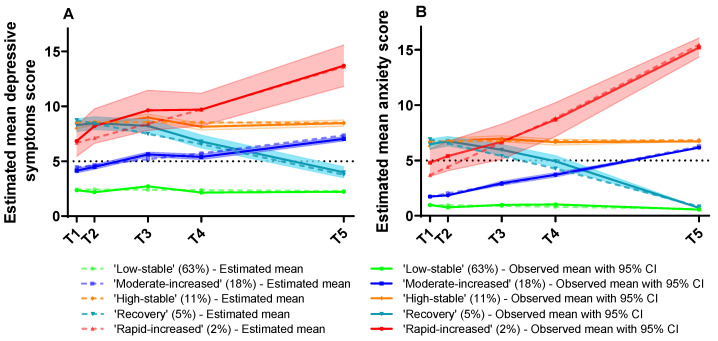
Simultaneous five-class trajectories of estimated mean scores and observed mean scores with a 95% CI of depressive symptoms (**A**) and anxiety (**B**) across the 17-month study timeline. T1: 3–10 February 2020; T2: 24 March to 3 April 2020; T3: 1–15 June 2020; T4: 10 September to 17 October 2020; T5: 10–18 June 2021.

Figure 2 provides a graphical representation of the five classes over time. The following classes were defined by the most parsimonious five-class model according to their trajectory patterns: (i) low–stable; (ii) moderate–increased; (iii) rapid–increased; (iv) recovery; and (v) high–stable. The largest trajectory group was the “Low–stable” group (64.3%, *n* = 8680), which included students who consistently showed low and stable levels of depressive symptoms and anxiety scores. The second largest group, the “Moderate–increased” group (18.2%, *n* = 2454), included students whose scores were low at the beginning of the pandemic and then increased to a moderate level. The third trajectory group, the “High–stable” group (11.1%, *n* = 1503), included students whose scores were high and stable. The fourth trajectory group, “Recovery” (4.5%, *n* = 609), included students whose scores were high at the beginning of the pandemic and then steadily decreased to a low score. The final trajectory group, “Rapid–increased” (1.8%, *n* = 248), included students whose scores were relatively low at the beginning of the pandemic and then rapidly increased to a higher level.

Table 3 shows the results of the multinomial logistic regression model using the ‘low–stable’ trajectory as the reference group. Participants with factors including study pressure, feelings of loneliness, despair during the lockdown, and sleep problems at the beginning of the pandemic were less likely to have ‘low–stable’ depressive symptoms and an anxiety trajectory. Meanwhile, participants with a higher levels of perceived social support were associated with an increased likelihood of belonging to the ‘low–stable’ trajectory. The odds of a ‘high stable’ trajectory were greater than those of a ‘low–stable’ trajectory for people who were older, used alcohol, had a history of psychological counselling, worried about being infected with COVID-19, were exposed to social media for more than one hour per day, had conflict with parents, were devastated by a romantic relationship breakup during the lockdown, and had low family functioning. The odds of a ‘moderate–increased’ trajectory were greater than those of the ‘low–stable’ trajectory for people who were female, had conflicts with parents, and had low family functioning. The odds of a ‘recovery’ trajectory were greater than those of the ‘low–stable’ trajectory for people with social media exposure of more than one hour per day, who did not believe COVID-19 can be protected at the beginning of the pandemic and who had low family functioning. The odds of a ‘rapid–increased’ trajectory were greater than for a ‘low–stable’ trajectory for people who were male and were devastated by a romantic relationship breakup during the lockdown.

Using the recovery group as the reference group, the results show that the ‘high–stable’ groups were characterized by more females, faced more worry about becoming infected with COVID-19, had more family conflict, were less devastated by a breakup, and endured less feelings of loneliness. The ‘moderate–increased’ group were characterized by more females, more sleep problems at the beginning of the pandemic, less history psychological consulting history before the pandemic, less study pressure, less devastation caused by a romantic relationship breakup during the lockdown, and fewer negative emotions (feelings of loneliness and devastation). See the online Supplementary Materials Section S3, Table S1, for full details.

## 4. Discussion

The study tracked the 16-month trajectories of anxiety and depressive symptoms among 13,494 Chinese university students enrolled in autumn 2019, with five observed time points. Students experienced an epidemic spike, a lockdown, normal life under routine prevention, a lockdown when the epidemic resumed, and a return to normal life. The results suggest that both depression symptoms and anxiety increased slightly over the 16 months among new students, with small but significant slopes, which is consistent with previous studies [10,39]. While the mean slopes of depression symptoms and anxiety were significantly higher in the 7 months after the pandemic outbreak than in the 16 months, this showed that the increase in mental health problems was slowing down. This supports the idea that the local outbreak of the pandemic did not have a serious negative impact on students’ mental health in the short term. Previous studies have found that the mental health status can recover from the distress reaction three or four months after the outbreak [11]; however, we did not find an immediate mental health reaction after the local recurrence of a pandemic. A possible explanation for this finding is that, unlike during the initial breakout, students were asked to stay in school instead of staying at home after the local recurrence. Long-term isolation at home could increase loneliness and stress [10,40], especially for students with low-functioning families. However, by staying in university, students would have peer support and the ability to meet their teachers face to face every day, with social support having a positive effect on students’ wellbeing [23,41].

In addition, the parallel trajectories of change in anxiety and depressive symptoms identified five heterogeneous groups: consistently low mental health problems—low–stable (64.3%), slowly increasing moderate mental health problems—moderate–increased (18.2%), constantly high mental health problems—high–stable (11.1%), high at start of the pandemic then steadily decreasing to a low score—recovery (4.5%), and rapidly increasing mental health problem profile—rapid–increased (1.8%). Most participants (64.3%) were resilient to the impact of the pandemic, showing a low and steady trajectory of anxiety and depressive symptoms. This was also consistent with previous findings which demonstrate that although some people may experience long-term distress following adverse events, resilience (maintaining healthy outcomes or ‘bouncing back’ after such events) is the most common and consistently observed response. [13,15,42,43]. Contrary to the resilient group’s low score trajectory, about 11% of participants suffer from a high score trajectory, which other studies [13,42] have called the chronic group. It was one of the most common groups identified by trajectory-based approaches followed by potentially traumatic events (PTEs), with previous studies identifying a mean prevalence of this group around 10% [42], which is similar to our findings.

Our results refined previous studies which reported that mental health problems worsened during the COVID-19 pandemic [8,39], particularly in the moderate–increased and rapid–increased student groups, which together account for 20% of participants. The mean prevalence rate of the increased group was 10% in previous studies [42]; however, compared with research during COVID-19, the prevalence rate of the increased group was 28% in the UK [13] and 10% in Australia [15]. A previous study indicated that the increased group was often associated with a lower levels of social support and involved exposure to pre-event and post-event stressors, and the prevalence rate was inconsistent [42]. On the other hand, about 5% of participants recovered their symptom levels over time, which is lower than 8.6% in the UK adult sample [13] and 8–9% in the Australian adult sample [15] (both of which collected data at the early stage of the pandemic). These results were also lower than a review of n = 54 studies globally which found a mean prevalence of 23% in the recovery group [42]. This may be because the participants in this study were young adults with a lower financial burden and their general health was better before the pandemic compared to in the population-based sample [44].

In this longitudinal study, we aimed to elucidate the temporal relationships between various covariates and mental health trajectories. Overall, these two multiple logistic regression models provide some evidence to support previous research, suggesting that some life stressors may have a negative impact on mental health trajectories. These include academic pressure, interpersonal conflict, lovelorn, and family conflict. Specifically, academic stress showed a higher prevalence in these four groups. Compared to the ‘low–stable group’, academic stress was more likely to lead to a deterioration in mental health trajectories, in line with the results of another Chinese study [45]. Other life events such as interpersonal conflicts and break-ups were also associated with high–stable and increased groups, suggesting that we need to accomplish more to help students with interpersonal problems during the pandemic. It is interesting to note that the high–stable group was associated with more family conflict and less devastation from separation compared to the recovery group, suggesting that conflict between students and their parents may have a more long-term negative psychological impact on students compared to other conflicts. Previous studies have found that family conflict is associated with higher depressive symptoms, anxiety, and GPA among Chinese undergraduates [46,47]. Chinese parents are known to have high expectations for their children in terms of academic achievement [48]. A semester of isolation and studying at home may increase parents’ anxiety about their child’s studies and may increase family conflict.

During the lockdown, feelings of loneliness and despair were also higher for participants in all other trajectory groups compared to the low–stable group, but compared to the recovery group, both the high–stable group and increased group had lower feelings of loneliness. This means feelings of loneliness may not be a fundamental factor in poorer mental health outcomes compared to other factors. Studies have shown that there is a strong link between social isolation and loneliness with poorer mental health outcomes in older populations [6]. However, mixed results have been found in young adults [6,21]. First, loneliness motivates people to look for ways to relieve this feeling, e.g., by investing in a new environment, seeking new relationships, and finding something to do. At this point, feelings of loneliness may subside. However, we should not forget that the core of loneliness is the response to whether there is a beneficial social relationship, which not only involves a judgment of the present but also an expectation of the future [49]. This finding therefore again shows the contradictory mechanism of loneliness. Because of loneliness, young adults invest in a new adaptive behavior, which may increase social communication, but may also lead to new mental health problems. Therefore, although it is conducive to adaptability to participate in society because of loneliness, there are risks in this process. In Chinese tradition, family relationships are the starting point or foundation for beneficial social relations. While this relationship conflict cannot be eliminated, young people may develop more self-protection mechanisms and negative behaviors which further affect their engagement in social communication. When comparing against the recovery group, we can see that family relationships still play an important role for young people. Family members or parents may be more influential than what the college students have imagined. As cross-cultural research has pointed out, Eastern cultures tend to have very close mother–child relationships. These children are less likely to suffer the negative effects of such relationships compared to children in the West [50].

Somatic factors at the early stage of the pandemic were also strongly associated with the different trajectories compared to the low–stable group, possibly reflecting a long-lasting sociocultural factor of Chinese people who tend to express depression in a somatic way [51,52].

Gender differences were also identified, with results showing that male students' mental health deteriorated faster than that of female students. This may be partly due to their negative attitudes towards emotional openness. As a result, they may be reluctant to use mental health services that are available to them during their studies [53]. Concurrently, females are more likely to have a higher level of depressive symptoms and anxiety than males, which has also been reported by other researchers [13,53].

Another notable finding is that, in contrast to alcohol consumption, smoking has not been found to be associated with the trajectory group. One reason could be that occasional smoking may initially be used to alleviate symptoms, and it takes time to identify the impact of smoking on mental health, but our follow-up period was not long enough to detect worsening symptoms [54].

## 5. Conclusions

To our knowledge, this is one of the first longitudinal research projects to evaluate mental health status from the outbreak of the COVID-19 pandemic to the lockdown, followed by regular prevention and local outbreaks among new undergraduate students. Preliminary findings from this study provide some important insights into the stringent pandemic preparedness measures on the mental health states of undergraduate students. The factors associated with class membership may provide insight into how future mental health education may be delivered. One example is the provision of more effective and personalized mental health education, such as smaller classes, as well as selective courses focusing on specific factors, e.g., courses to improve family relationship and courses to improve relationships. Providing more social interaction activities, such as online peer support groups, is also helpful.

There are a few limitations that should be considered when interpreting these results. First, our sample may not be fully representative of the general population of university students in China, as it was collected through online sampling. For example, the proportion of females in the sample was relatively high. This may limit the generalizability of our results. Second, the first survey may not have provided a genuine baseline measure of mental health status, as public health restrictions such as the Wuhan lockdown were imposed 10 days before the first survey. Third, mental health status was collected by self-report scales, and although the PHQ-9 and GAD-7 are robust measures, clinician-administered diagnostic interviews may have yielded different results.

Future research needs to continue to track the long-term psychological effects of the COVID-19 pandemic and compare the different subsequent mental health outcomes from different prevention strategies to prepare us for other challenges ahead.

The present study found a slight increase in the mental health status of new undergraduate students in China during the 16 months of the pandemic. The local outbreak did not have any serious short-term negative impact on students’ mental health. Less than one-third of new undergraduate students experienced deteriorated or constantly high depressive symptoms and anxiety throughout the study period, 5% of students’ mental health status improved, while the rest of students were resilient during this period. Trajectory groups are associated with environmental factors, negative emotions, somatic factors, and social factors. Mental health staff in colleges should be aware that students with severe conflicts with other family members and less social support may need more attention, and students with long-lasting sleep problems may be more vulnerable, as well as those with a drinking problem and other mental health problems prior to the crisis. Thus, it is important to reform mental health education in colleges from mass education to personalized education and make it easier to receive online mental health support during the COVID-19 pandemic and future crises.

## Figures and Tables

**Table 1:** Detection rates of depression and anxiety in T1–T5.

Variables	Gender	T1		T2		T3		T4		T5	
		*n*	*%*	*n*	*%*	*n*	*%*	*n*	*%*	*n*	*%*
Probable clinical depression	Male	634	20.60%	850	26.10%	1144	31.70%	1021	26.90%	846	28.60%
	Female	1697	21.40%	2138	24.80%	2698	31.20%	2583	28.10%	2519	32.20%
	Total	2331	21.20%	2988	25.10%	3842	31.40%	3604	27.70%	3365	31.20%
Probable clinical anxiety	Male	344	11.20%	474	14.50%	663	18.40%	726	19.10%	561	19.00%
	Female	797	10.10%	1128	13.10%	1367	15.80%	1714	18.60%	1557	19.90%
	Total	1141	10.40%	1602	13.50%	2030	16.60%	2440	18.80%	2118	19.70%

**Table 2:** Model fit indices and estimated class sizes for the growth mixture model.

No.	AIC	BIC	BLRT	LMRT	Entropy	Sample Size Per Class Based on Most Likely Class Members
1	567,385.13	567,565.37	-	-	-	13,494
2	563,495.35	563,713.14	*p* < 0.001	*p* < 0.001	0.877	2313/11,181
3	559,355.13	559,610.47	*p* < 0.001	*p* < 0.001	0.905	278/9323/3893
4	557,998.35	558,291.24	*p* < 0.001	*p* < 0.001	0.870	9265/272/1401/2556
5	556,228.92	556,559.36	*p* < 0.001	*p* < 0.001	0.882	1503/2454/609/248/8680
6	554,851.71	555,219.70	*p* < 0.001	*p* = 0.2332	0.895	8501/753/2116/132/1764/228

AIC, the Akaike information criterion; BIC, the Bayesian information criterion; BLRT, the bootstrap likelihood ratio test; LMRT, the Lo–Mendell–Rubin likelihood ratio test. The bold line indicates the model chosen as the overall best-fitting class.

**Table 3:** Predictors (odds ratios) of depressive symptoms and anxiety trajectories.

	High–Stable	Moderate–Increased	Recovery	Rapid–Increased
Age	1.21 *** (1.10, 1.34)	1.05 (0.97, 1.13)	1.13 (0.96, 1.33)	1.04 (0.84, 1.28)
Gender (male = reference)				
Female	1.10 (0.86, 1.41)	1.44 ** (1.18, 1.76)	0.97 (0.67, 1.39)	0.58 ** (0.38, 0.89)
Living place (rural areas = reference)				
Live in city	1.08 (0.88, 1.33)	1.02 (0.87, 1.20)	0.96 (0.70, 1.31)	1.34 (0.90, 2.00)
Daily physical exercise time (less than 1 h = reference) ^†^				
More than 1 h	1.08 (0.93, 1.26)	1.00 (0.89, 1.12)	0.83 (0.66, 1.05)	0.86 (0.64, 1.14)
Smoking (Never = reference)				
Ever	1.05 (0.65, 1.72)	0.90 (0.56, 1.43)	1.77 (0.97, 3.22)	0.93 (0.41, 2.13)
Alcohol intake (Never = reference)				
Ever	1.33 * (1.05, 1.67)	1.17 (0.98, 1.40)	0.93 (0.66, 1.31)	1.17 (0.74, 1.85)
Pre-existing physiological health condition (no condition = reference)				
Yes	1.07 (0.32, 3.61)	0.43 (0.08, 2.22)	1.22 (0.24, 6.11)	1.38 (0.27, 7.04)
Pre-existing mental health condition (no condition = reference)				
Yes	0.47 (0.15, 1.49)	0.81 (0.22, 3.01)	0.71 (0.16, 3.08)	1.33 (0.26, 6.66)
Psychological consulting history (no = reference)				
Yes	1.91 * (1.23, 2.97)	1.05 (0.68, 1.64)	2.00 (1.08, 3.71)	2.46 (1.29, 4.69)
Worry about family members becoming infected with COVID-19 (no = reference)				
Yes	0.85 (0.53, 1.37)	1.00 (0.74, 1.35)	2.05 (1.10, 3.82)	0.87 (0.45, 1.69)
Worry about oneself becoming infected with COVOD-19 (no = reference)				
Yes	2.63 ** (1.74, 3.99)	1.13 (0.87, 1.49)	0.88 (0.56, 1.39)	0.91 (0.50, 1.66)
Daily social media exposure (less than 1 h = reference)				
More than 1 h	1.36 * (1.11, 1.68)	1.11 (0.95, 1.30)	1.60 * (1.17, 2.19)	1.26 (0.83, 1.93)
Whether believe COVID-19 protection measures are effective (no = reference)				
Yes	1.28 (0.69, 2.39)	0.75 (0.48, 1.18)	0.48 ** (0.24, 0.94)	0.85 (0.32, 2.25)
Whether implemented COVID-19 preventive measures (no = reference)				
Yes	0.73 (0.47, 1.14)	1.24 (0.79, 1.94)	1.00 (0.50, 2.02)	1.19 (0.48, 2.97)
Study pressure during the lockdown (no = reference) ^‡^				
Yes	6.32 *** (4.58, 8.71)	2.40 *** (2.02, 2.84)	2.31 ** (1.63, 3.28)	4.87 * (2.66, 8.93)
Conflicts with parents during the lockdown (no = reference) ^‡^				
Yes	2.04 *** (1.66, 2.52)	1.43 *** (1.22, 1.68)	1.42 (1.04, 1.96)	1.23 (0.82, 1.86)
Devastated by a breakup romantic relationship during the lockdown (no = reference) ^‡^				
Yes	1.66 * (1.15, 2.39)	1.42 (1.03, 1.96)	1.63 (0.94, 2.84)	3.54 ** (2.11, 5.93)
Conflicts with teachers or classmates during the lockdown (no = reference) ^‡^				
Yes	2.66 ** (1.68, 4.20)	1.55 (0.96, 2.51)	1.37 (0.60, 3.11)	2.78 (1.38, 5.61)
Ever feel loneliness during the lockdown (no = reference) ^§^				
Yes	4.98 *** (3.42, 7.24)	3.16 *** (2.15, 4.63)	4.39 ** (2.62, 7.36)	9.44 ** (5.61, 15.89)
Ever feel despair during the lockdown (no = reference) ^§^				
Yes	8.59 *** (5.61, 13.14)	3.89 ** (2.55, 5.94)	5.90 ** (3.29, 10.57)	9.57 ** (5.35, 17.11)
Sleeping problem (no = reference)				
Yes	8.74 *** (6.35, 12.01)	2.57 ** (1.80, 3.67)	10.68 *** (7.15, 15.95)	5.38 ** (3.16, 9.17)
Perceived social support score	0.96 *** (0.95, 0.97)	0.99 * (0.98, 1.00)	0.97 *** (0.95, 0.98)	0.96 *** (0.93, 0.98)
Family functioning score ^†^	0.94 ** (0.90, 0.99)	0.96 * (0.93, 1.00)	0.87 *** (0.82, 0.93)	0.91 (0.83, 1.01)

Note: * *p* < 0.05, ** *p*< 0.01, *** *p* < 0.001. The reference class included ‘low–stable’ depressive symptoms and anxiety trajectories. ^†^ collected at T2, ^‡^ collected at T3, ^§^ collected at T4, while the other variables were collected at T1.

## Data Availability

The database is not available for direct access, but can be requested from researchers.

## Supplementary Materials

The following supporting information can be downloaded at: https://www.mdpi.com/article/10.3390/ijerph20065024/s1. Supplementary Materials Section S1: Details of covariant variables. Supplementary Materials Section S2: Statistical analysis in detail. Supplementary Materials Section S3: Results of the multinomial logistic regression model using the ‘recovery’ trajectory as the reference group, Table S1: Predictors (odds ratios) of depressive symptoms and anxiety trajectories.
