# Supplementary material for: An Unusual College Experience: 16-Month Trajectories of Depressive Symptoms and Anxiety among Chinese New Undergraduate Students of 2019 during the COVID-19 Pandemic
Source: Int J Environ Res Public Health. 2023 Mar 12;20(6):5024. doi: 10.3390/ijerph20065024 (PMC10048813; doi:10.3390/ijerph20065024)
Supplement: Supplementary file 1 [file ijerph-20-05024-s001.zip › ijerph-2226003-supplementary.pdf]

Online supplementary material for 'An Unusual College Experience: 16 Months Trajectories of Depressive Symptom and Anxiety of Chinese New Undergraduate Students during the COVID-19'

This online supplement contains further details of covariant variables used in the regression analyses, the syntax of statistical analysis, and the result of the multinomial logistic regression model using the 'recovery' trajectory as the reference group.

Section S1 Details of covariant variables

A series of covariant variables were extracted as follows:

1.1 Characteristics covariates

age (years)

gender (female=1; male = 0)

Living place

Item: Where your family lives? (0 = rural area, 1= small town or county, small and medium-sized cities, big cities)

1.2 Life style covariates

Daily physical exercise time

Item: During the past two weeks, the average daily time spent on exercise was: (0= less than 1 hour, 1 = 1-2 hours, 3-5 hours, more than 5 hours).

Smoking

Item: Do you smoke? (0 = never, 1 = already quit, sometimes, 1-5 cigarettes per day, 6-10 cigarettes per day, more than 10 cigarettes per day).

Alcohol intake

Item: Do you drink? (0 = never, 1 = already quit, sometimes, less than 100ml per day, 150-250ml per day, more than 250ml per day).

1.3 pre-COVID-19 health covariates

Pre-existing physiological health condition

Item: Do you have a major physical illness? (0 = no, 1 = yes).

Pre-existing mental health condition

Item: Have you ever been diagnosed with a mental illness? (0 = no, 1 = yes).

Psychological consulting history

Item: Have you received psychological counseling services from a professional (counselor, psychiatrist, etc.) in the past? (0 = no, 1 = yes).

1.4 COVID-19 related covariates

Worry about family members becoming infected with COVID-19

Item: Do you worry about your family members becoming infected with COVID-19? (0 = not worried 1 = worried, very worried)

Worry about oneself becoming infected with COVOD-19

Item: Do you worry about yourself becoming infected with COVID-19? (0 = not worried 1 = worried, very worried)

Daily social media exposure

Item: The time of day you follow information about COVID-19 is (0= less than 1 hour, 1 = 1-2 hours, 3-5 hours, more than 5 hours)

Whether believe COVID-19 can be protected

Item: Do you think COVID-19 can be prevented? (0 = no, 1 = yes)

Whether implemented COVID-19 preventive measures

Have you implemented the precautions you have learned since the COVID-19 outbreak? (0 = no, 1 = yes, sometime; yes, severe)

1.5 Environmental covariates

Study pressure during the lockdown

Item: Have you recently felt overwhelmed by the increased pressure to study because of a large drop in your grades, or a failure in an exam, or inefficiency in your studies? (0 = Basically no, 1 = yes, sometime; yes, severe)

Conflicts with parents during the lockdown

Item: Have you had any recent conflicts with your parents? (0 = Basically no, 1 = yes, sometime; yes, severe)

Devastated by a breakup romantic relationship during the lockdown

Item: Have you recently been hit hard by a breakup or a rejection of a confession of love? (0 = Basically no, 1 = yes, sometime; yes, severe)

Conflicts with teachers or classmates during the lockdown

Item: Have you recently had a conflict with a teacher or classmate that strained your relationship? (0 = Basically no, 1 = yes, sometime; yes, severe)

1.6 Negative emotions covariates

Ever feel loneliness during the lockdown

Item: How often did you feel lonely during the lockdown? (0 = never, occasionally, 1 = frequently, nearly every day)

Ever feel despair during the lockdown

Item: In the last month, do you feel desperate about your situation? (0 = no, 1 = yes)

1.7 Somatic covariates

Sleeping problem

The total score of the Youth Self-Rating Insomnia Scale (YSIS) (Liu et al., 2019).

1.8 Social covariates

Perceived social support score

The total score of the Multidimensional Scale of Perceived Social Support (Zimet, Dahlem, Zimet, & Farley, 1988).

Family function

The total score of Family APGAR (Smilkstein, Ashworth, & Montano, 1982).

Section S2 Statistical analysis detail

2.1 Detail of the GMM

Due to the time intervals of five surveys are not equidistant, and the assessment period of each time was around a month (see more detail at 2.1), we set T1 as the initial status and time score of T1 for the model set to 0, and time scores of Tx are defined as the number of months between the assessment time of T1 and Tx, such as T3 was assessed at June 1—15, which was 4 month after T1 (February 3--10), so time score of T3 was set to 4 (Time score of T1 = 0, T2 = 1, T3 = 4. T4 = 7, T5 = 16) (Muthén & Muthén, 1998-2017).

2.2 One group GMM model in Mplus

```
TITLE: GMM FOR ONE GROUP
DATA:
   FILE IS T1-T5.CSV;
VARIABLE:
   NAMES ARE PHQ_1 PHQ_2 PHQ_3 PHQ_4 PHQ_5
   GAD_1 GAD_2 GAD_3 GAD_4 GAD_5 X1-X24;
   USEVARIABLES ARE PHQ_1 PHQ_2 PHQ_3 PHQ_4 PHQ_5
   GAD_1 GAD_2 GAD_3 GAD_4 GAD_5;
   CLASSES = C (1);
   MISSING = PHQ_1 PHQ_2 PHQ_3 PHQ_4 PHQ_5
   GAD_1 GAD_2 GAD_3 GAD_4 GAD_5 (99);
   AUXILIARY=X1-X24;
ANALYSIS: TYPE = MIXTURE;
   STARTS=100 80;
   PROCESSOR=8;
MODEL:
   %OVERALL%
    I S| PHQ_1@0 PHQ_2@1 PHQ_3@4 PHQ_4@7 PHQ_5@16;
    I_2 S_2| GAD_1@0 GAD_2@1 GAD_3@4 GAD_4@7 GAD_5@16;
OUTPUT:
PLOT:
         TYPE IS PLOT3;
         SERIES=PHQ_1 PHQ_2 PHQ_3 PHQ_4 PHQ_5(*);
         SERIES=GAD_1 GAD_2 GAD_3 GAD_4 GAD_5(*);
```

2.3 Five groups GMM model in Mplus

The only difference between one group and the other group is START, we set START 800 10 at first, and find the best loglikelihood value has been replicated, then we change START to 1600 20 to make sure the best loglikelihood value stays the

same(Muthén & Muthén, 1998-2017). We use the same way for class 2 through class 6. The syntax below was for class 5. We did not set output in this stage because BLRT can be boosted by adding "optseed" of the best loglikelihood value(Asparouhov & Muthén, 2012), so we estimated BLRT and LMRT in the next stage.

```
TITLE: GMM FOR FIVE GROUP
DATA:
  FILE IS T1-T5.CSV;
VARIABLE:
  NAMES ARE PHQ_1 PHQ_2 PHQ_3 PHQ_4 PHQ_5
  GAD_1 GAD_2 GAD_3 GAD_4 GAD_5 X1-X24;
  USEVARIABLES ARE PHQ_1 PHQ_2 PHQ_3 PHQ_4 PHQ_5
  GAD_1 GAD_2 GAD_3 GAD_4 GAD_5;
  CLASSES = C (5);
  MISSING = PHQ_1 PHQ_2 PHQ_3 PHQ_4 PHQ_5
  GAD_1 GAD_2 GAD_3 GAD_4 GAD_5 (99);
  AUXILIARY=X1-X24;
ANALYSIS: TYPE = MIXTURE;
  STARTS=1600 20;
  PROCESSOR=8;
MODEL:
  %OVERALL%
   I S| PHQ_1@0 PHQ_2@1 PHQ_3@4 PHQ_4@7 PHQ_5@16;
   I_2 S_2| GAD_1@0 GAD_2@1 GAD_3@4 GAD_4@7 GAD_5@16;
OUTPUT:
PLOT:
      TYPE IS PLOT3;
      SERIES=PHQ_1 PHQ_2 PHQ_3 PHQ_4 PHQ_5(*);
      SERIES=GAD_1 GAD_2 GAD_3 GAD_4 GAD_5(*);
```

2.3.1 Estimate five group's BLRT and LMRT

Based on the optimum seed for best loglikelihood value in the previous stage's output, we add OPTSEED=856612 to the syntax and along with OUTPUT: TECH11 TECH14 to estimate BLRT and LMRT for class 5. Also, use SAVEDATA: FILE= C5.CSV; SAVE=CPROB to save class information and AUXILIARY variables for next stage multinomial logistic regression.

```
TITLE: GMM FOR FIVE GROUP BLRT and LMRT
DATA:
  FILE IS T1-T5.CSV;
VARIABLE:
  NAMES ARE PHQ_1 PHQ_2 PHQ_3 PHQ_4 PHQ_5
  GAD_1 GAD_2 GAD_3 GAD_4 GAD_5 X1-X24;
```

```
  USEVARIABLES ARE PHQ_1 PHQ_2 PHQ_3 PHQ_4 PHQ_5
  GAD_1 GAD_2 GAD_3 GAD_4 GAD_5;
  CLASSES = C (5);
  MISSING = PHQ_1 PHQ_2 PHQ_3 PHQ_4 PHQ_5
  GAD_1 GAD_2 GAD_3 GAD_4 GAD_5 (99);
  AUXILIARY=X1-X24;
ANALYSIS: TYPE = MIXTURE;
  STARTS=0 0;
  PROCESSOR=8;
  OPTSEED=856612;
MODEL:
   %OVERALL%
   I S| PHQ_1@0 PHQ_2@1 PHQ_3@4 PHQ_4@7 PHQ_5@16;
   I_2 S_2| GAD_1@0 GAD_2@1 GAD_3@4 GAD_4@7 GAD_5@16;

OUTPUT: TECH11 TECH14;
SAVEDATA: FILE= C5.CSV; SAVE=CPROB;
PLOT:
        TYPE IS PLOT3;
        SERIES=PHQ_1 PHQ_2 PHQ_3 PHQ_4 PHQ_5(*);
        SERIES=GAD_1 GAD_2 GAD_3 GAD_4 GAD_5(*);
```

2.4 Multinomial logistic regression

According to Asparouhov and Muthén (2014), multinomial logistic regression was estimated based on logits for the classification probabilities for the most likely latent class membership by previous stage's output. The output command tech11 will give results for different reference groups; CINTERVAL will estimate 95% CI.

```
TITLE:   Multinomial logistic regression
DATA:
  FILE IS C5.CSV;
VARIABLE:
  NAMES ARE PHQ_1 PHQ_2 PHQ_3 PHQ_4 PHQ_5 GAD_1
   GAD_2 GAD_3 GAD_4 GAD_5 ID X1-X23 P1-P13 N;
  USEVARIABLES ARE X1-X23 N;
   CLASSES = C(5);
   NOMINAL=N;
   MISSING PHQ_1 PHQ_2 PHQ_3 PHQ_4 PHQ_5 GAD_1
    GAD_2 GAD_3 GAD_4 GAD_5 X2-X27 (99);

ANALYSIS: TYPE = MIXTURE; STARTS = 0;
```

```
MODEL: %OVERALL%
          C ON X1-X23;
    %c#1%
[n#1@4.57];
[n#2@2.372];
[n#3@0.707];
[n#4@-0.128];

%c#2%
[n#1@-0.688];
[n#2@2.135];
[n#3@-4.566];
[n#4@-2.632];

%c#3%
[n#1@-0.581];
[n#2@-2.88];
[n#3@1.686];
[n#4@-6.019];

%c#4%
[n#1@4.23];
[n#2@4.548];
[n#3@-1.903];
[n#4@6.496];

%c#5%
[n#1@-6.638];
[n#2@-3.988];
[n#3@-4.875];
[n#4@-9.309];


output:
tech11; CINTERVAL;
```

Section S3 Result of the multinomial logistic regression model using 'recovery' trajectory as the reference group

Table S1. Predictors (odds ratios) of depressive symptoms and anxiety trajectories

	High-stable	Moderate-increased	Rapid-increased	Low-stable
Age	1.17(0.94,1.46)	1.01(0.81,1.27)	1.09(0.85,1.40)	0.97(0.78,1.20)
Gender (male = reference)				
Female	1.89*(1.20,2.98)	2.47*(1.56,3.92)	1.66(1.00,2.77)	1.72(1.12,2.63)
Living place (rural areas = reference)				
Live in city	0.81(0.53,1.24)	0.76(0.50,1.16)	0.72(0.45,1.15)	0.75(0.50,1.11)
Daily physical exercise time (less than 1 hour = reference) a				
More than 1 hour	1.26(0.93,1.70)	1.17(0.86,1.58)	0.97(0.69,1.36)	1.17(0.87,1.55)
Smoking (Never = reference)				
Ever	1.13(0.47,2.71)	0.96(0.38,2.42)	1.90(0.77,4.70)	1.07(0.47,2.45)
Alcohol intake (Never = reference)				
Ever	1.13(0.70,1.84)	1.00(0.62,1.62)	0.80(0.47,1.35)	0.86(0.54,1.35)
Pre-existing physiological health condition (no condition = reference)				
Yes	0.78(0.12,4.92)	0.31*(0.03,2.88)	0.88(0.13,5.93)	0.72(0.14,3.68)
Pre-existing mental health condition (no condition = reference)				
Yes	0.36*(0.07,1.77)	0.61(0.09,4.44)	0.53(0.10,2.91)	0.76(0.15,3.80)
Psychological consulting history (No = reference)				
Yes	0.78(0.40,1.51)	0.43***(0.21,0.90)	0.81(0.38,1.73)	0.41***(0.21,0.77)
Worry about family members becoming infected with COVID-19 (No = reference)				
Yes	0.98(0.46,2.11)	1.15(0.56,2.36)	2.36(1.02,5.47)	1.15(0.59,2.25)
Worry about oneself becoming infected with COVOD-19 (No = reference)				
Yes	2.90(1.46,5.76)	1.25(0.66,2.38)	0.97(0.49,1.92)	1.10(0.60,2.01)
Daily social media exposure (Less than 1 hour = reference)				
More than 1 hour	1.08(0.69,1.69)	0.88(0.56,1.37)	1.26(0.77,2.06)	0.79(0.52,1.21)
Whether believe COVID-19 can be protected (No = reference)				
Yes	1.52(0.54,4.29)	0.89(0.32,2.46)	0.56(0.19,1.67)	1.18(0.44,3.16)
Whether implemented COVID-19 preventive measures (No = reference)				
Yes	0.61(0.24,1.58)	1.04(0.39,2.76)	0.84(0.30,2.37)	0.84(0.34,2.08)
Study pressure during the lockdown (No = reference) ‡				
Yes	1.30(0.66,2.56)	0.49**(0.26,0.92)	0.48**(0.24,0.93)	0.21***(0.11,0.38)
Conflicts with parents during the lockdown (No = reference) ‡				
Yes	1.66*(1.07,2.57)	1.16(0.76,1.79)	1.16(0.71,1.88)	0.81(0.54,1.22)
Devastated by a breakup romantic relationship during the lockdown (No = reference) ‡				
Yes	0.47***(0.27,0.81)	0.40***(0.23,0.70)	0.46***(0.24,0.89)	0.28***(0.17,0.48)
Conflicts with teachers or classmates during the lockdown (No = reference) ‡				
Yes	0.96(0.49,1.86)	0.56*(0.27,1.16)	0.49*(0.20,1.19)	0.36***(0.18,0.72)
Ever feel loneliness during the lockdown (No = reference) §				
Yes	0.53***(0.32,0.86)	0.33***(0.20,0.57)	0.47***(0.26,0.83)	0.11***(0.06,0.18)
Ever feel despair during the lockdown (No = reference) §				
Yes	0.90(0.53,1.51)	0.41***(0.23,0.72)	0.62*(0.34,1.12)	0.11***(0.06,0.19)
Sleeping problem (No = reference)				

Yes	1.62(0.96,2.74)	0.48***(0.27,0.84)	1.99(1.14,3.45)	0.19***(0.11,0.32)
Perceived social support score	1.01(0.98,1.03)	1.04**(1.01,1.06)	1.01(0.99,1.04)	1.05***(1.02,1.07)
Family function score †	1.03(0.93,1.14)	1.06(0.95,1.17)	0.96(0.86,1.07)	1.10(0.99,1.21)

Note: $^{*}p< 0.05$, $^{**}p< 0.01$, $^{***}p< 0.001$; The reference class is '**recovery**' depressive symptoms and anxiety trajectories.

† collected at T2, ‡ collected at T3, § collected at T4, other variables were collected at T1.

References

Asparouhov, Tihomir, & Muthén, Bengt. (2012). Using Mplus TECH11 and TECH14 to test the number of latent classes. *Mplus Web Notes, 14*.

Asparouhov, Tihomir, & Muthén, Bengt. (2014). Auxiliary Variables in Mixture Modeling: Three-Step Approaches Using Mplus. *Structural Equation Modeling: A Multidisciplinary Journal, 21*(3), 329-341. doi:10.1080/10705511.2014.915181

Liu, Xianchen, Yang, Yanyun, Liu, Zhen-Zhen, Luo, Yachen, Fan, Fang, & Jia, Cun-Xian. (2019). Psychometric properties of Youth Self-Rating Insomnia Scale (YSIS) in Chinese adolescents. *sleep and biological rhythms, 17*(3), 339-348. doi:10.1007/S41105-019-00222-3

Muthén, L.K., & Muthén, B.O. (1998-2017). *Mplus User's Guide* (Eighth Edition ed.).

Smilkstein, G., Ashworth, C., & Montano, D. (1982). Validity and reliability of the family APGAR as a test of family function. *J Fam Pract, 15*(2), 303-311.

Zimet, Gregory D., Dahlem, Nancy W., Zimet, Sara G., & Farley, Gordon K. (1988). The Multidimensional Scale of Perceived Social Support. *Journal of Personality Assessment, 52*(1), 30-41. doi:10.1207/s15327752jpa5201_2
